# First Experiences of Patients and Healthcare Professionals with Routine Use of Patient-Reported Outcome Measures for Venous Thromboembolism

**DOI:** 10.1055/a-2600-7707

**Published:** 2025-05-23

**Authors:** Cindy M.M. de Jong, Sophie N.M. ter Haar, Willem Jan W. Bos, Paul L. den Exter, Menno V. Huisman, Marlon H.C. Kosterink, Thijs E. van Mens, Frederikus A. Klok

**Affiliations:** 1 Department of Medicine, Thrombosis and Hemostasis, Leiden University Medical Center, Leiden, the Netherlands; 2 Department of Medicine, Nephrology, Leiden University Medical Center, Leiden, the Netherlands; 3 Department of Internal Medicine, St. Antonius Hospital, Nieuwegein, the Netherlands; 4 Department of Information Technology and Digital Innovation, Leiden University Medical Center, Leiden, the Netherlands

**Keywords:** venous thromboembolism, patient-reported outcome measures, patient-centered care, patient outcome assessment, implementation science

## Abstract

**Background:**

Venous thromboembolism (VTE) can considerably limit patients' functioning and quality of life. Using patient-reported outcome measures (PROMs), the full impact of VTE on individual patients can be captured.

**Methods:**

To evaluate the experiences of patients and healthcare professionals with the routine use of PROMs for VTE patients visiting the outpatient clinic, a mixed-methods study was performed at Leiden University Medical Center, the Netherlands. VTE PROMs were incorporated into routine care since March 2023, through a digital application sending patients invitations to complete PROMs. Quantitative and qualitative data were obtained from semi-structured interviews with patients and involved healthcare professionals. The NoMAD (normalization measure development) questionnaire was used to assess the implementation process from the professionals' perspective. Patients aged ≥18 years who experienced VTE and completed PROMs at two follow-up time points during ≥3 months follow-up and VTE patients who did not complete PROMs at both time points were asked to participate.

**Results:**

Eight patients (five completed PROMs; three did not) and four professionals were interviewed. Both patients and professionals experienced the use of PROMs as neutral to predominantly positive (lower limit 3 on a scale of 1–5). All professionals valued the effects of PROMs on their work. Most patients felt the questionnaires contained too many questions. Suggestions to improve the completion rate, accessibility, PROMs content, and the digital tool were shared.

**Conclusion:**

PROMs were believed to provide additional value during preparation for the appointment and during the consultation. The first experiences of patients and professionals, tending toward positive, can be used to improve PROMs application and support implementation in routine thrombosis care.

## Introduction

After experiencing deep vein thrombosis (DVT) or acute pulmonary embolism (PE), patients may encounter a wide spectrum of health effects and long-term consequences. 1 2 3 4 5 6 7 8 Venous thromboembolism (VTE) and its sequelae may affect both physical and psychosocial functioning, considerably limiting patients' ability to work, psychological well-being, and quality of life. 9 10 11 12 13 14 15 16 17 Assessment of patient-centered outcomes may therefore contribute to a better understanding of the impact of the venous thromboembolic event on individual patients, help guide the agenda for the consultation, and tailor management decisions to the patient's needs and values. Such outcomes can be measured using patient-reported outcome measures (PROMs). PROMs are standardized questionnaires that are completed by patients, to assess their symptom burden, perceived health status, and well-being, capturing outcomes of care and the impact of disease from the patient's perspective. 18 19 20 Routine use of PROMs could empower patients to make informed healthcare decisions. 18 21 Moreover, complementing traditionally measured clinical outcomes with patient-reported outcomes is an important step toward patient-centered health care. 22

To facilitate the use of patient-centered outcomes in daily clinical practice, the multidisciplinary ICHOM-VTE project (International Consortium for Health Outcomes Measurement project for VTE) established a standardized set of patient-relevant outcome measures for patients with VTE. 23 During a modified Delphi process, an international working group consisting of VTE experts as well as patient representatives selected the outcomes that were considered to matter most to patients. This set of outcomes along with recommended outcome measures, including PROMs, resulted from a thorough process of development with the engagement of patient representatives and was designed to apply to all patients diagnosed with VTE aged 16 years and older. The PROMs that are part of this core set of outcomes have been embedded in routine care at the thrombosis outpatient clinic of the Leiden University Medical Center (LUMC; the Netherlands). Important lessons can be learned from the implementation process and the first experiences of patients and healthcare professionals. The aim of this study was to assess the feasibility of PROMs completion and experiences with the routine use of PROMs for VTE patients treated in our center.

## Methods

### Setting

PROMs for adult VTE patients have been incorporated into our routine patient pathway since March 2023. During the implementation phase, PROMs based on the outcome measures that were selected during the ICHOM-VTE project ( Table 1 ) were implemented using a digital application (Brightfish), which is integrated into the electronic health records system. 23 With the use of this digital tool, an invitation link is sent to the patient by email ahead of the scheduled appointment at the outpatient clinic. The link leads the patient to an online page where the questionnaires can be completed. This allows the patients to fill out the PROMs at home before their visit to the outpatient clinic. All patients who experienced VTE and had a scheduled first appointment at the thrombosis outpatient clinic were sent an invitation link to complete PROMs.

**Table 1:** Patient-centered outcomes with patient-reported outcome measures, which are part of the ICHOM-VTE standardized set of outcomes

Patient-centered outcome	Patient-reported outcome measure
Quality of life	PROMIS Scale v1.2—Global Health PEmb-QoL questionnaire VEINES-QOL questionnaire
Functional limitations (including ability to work)	Post-VTE Functional Status Scale
Pain (including symptom severity)	PROMIS Short Form v2.0—Pain Intensity—3a
Dyspnea (including symptom severity)	PROMIS Short Form v1.0—Dyspnea Severity—10a
Psychosocial wellbeing	Patient Health Questionnaire (PHQ-9) Generalized Anxiety Disorder (GAD-7)
Satisfaction with treatment	Single question: “Are you satisfied with your VTE treatment?” If required: Anti-Clot Treatment Scale (ACTS)
Changes in life view	Single question: “Have you experienced a change in your expectations, aspirations, values, or perspectives on life opportunities since the diagnosis of VTE?”

Abbreviations: ICHOM, International Consortium for Health Outcomes Measurement; VTE, venous thromboembolism.

Note: The complete ICHOM set of patient-centered outcome measures for venous thromboembolism is available via https://www.ichom.org/. ^23^

**Fig. 1:**
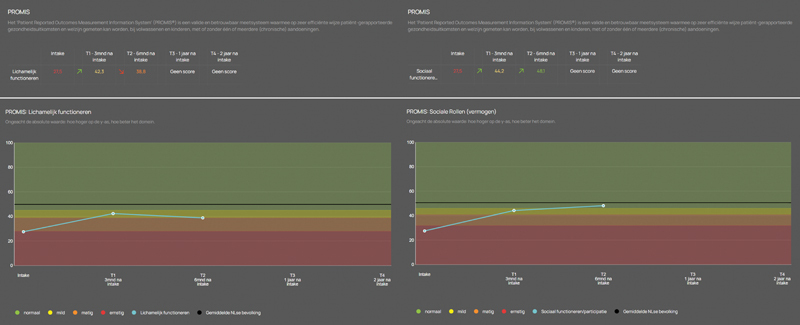
PROMs dashboard in the electronic medical records. Example of the dashboard in the electronic medical records (in the Dutch language), showing the summary of PROM results per questionnaire (above in the figure) along with a graphical display (below in the figure). The answers to each of the questions of the completed questionnaires can also be reviewed in the dashboard. Note: in our center, the PROMIS short form “Physical Function” (left in the figure) and short form “Ability to Participate in Social Roles and Activities” (right in the figure) were implemented, which contain additional questions about physical health and social activities and roles compared to the PROMIS short form “Global Health” to delve deeper into these domains. PROMs: patient-reported outcome measures.

PROM results are immediately visible in a dashboard within the electronic medical records facilitated by the embedded digital tool, displaying the results in an intuitive way ( Fig. 1 ). Healthcare professionals can access the dashboard to review the completed questionnaires and graphical display of PROM results, which helps to interpret the responses and visualizing the course of PROM results when multiple measurements become available during follow-up. The PROM results can be used to optimally prepare for the patient appointment, as well as to guide the conversation with the patient during the consultation.

The first invitation to complete PROMs is sent out to patients 1 week before the first follow-up contact, which is scheduled around 7 to 10 days following the VTE diagnosis according to the local patient pathway. Following the first measurement time point (T0), the PROMs are scheduled by the digital tool at fixed time points: patients receive invitations after 3 months (T1), after 6 months (T2), at 1-year follow-up (T3) and then yearly up to 3 years after the VTE diagnosis, for as long as the patient is under care. For the first time point (T0), the questionnaires could be answered 1 week before the first visit until 1 week after the visit. From the second time point (T1) onward, a 2-week window around the measurement time point was applied for the questionnaires to be open.

### Design

The objective of this study was to evaluate the feasibility of the completion and application of PROMs and experiences with the use of PROMs in routine care for patients with VTE visiting our outpatient clinic. We aimed to assess the experiences of both patients and involved healthcare professionals. Evaluation of the scores and results of the PROMs was not within the scope of the current study.

A mixed-methods study was performed utilizing both quantitative and qualitative data. Quantitative data were obtained from statistics recorded by the digital PROMs tool, from 5-point Likert scale questions applied in semi-structured interviews with patients and healthcare professionals, and from the NoMAD (normalization measure development) questionnaire completed by healthcare professionals. 24 25 Qualitative data were obtained from semi-structured interviews with patients and healthcare professionals. The Institutional Review Board of the LUMC approved the study (protocol 132775).

### Participants

Patients aged 18 years and older who were diagnosed with acute PE and/or DVT of the lower or upper extremity and received follow-up for at least 3 months at the outpatient clinic were identified in September 2023 based on scheduled appointments. Patients who completed PROMs at the first two time points (around 7 to 10 days after VTE diagnosis [T0] and after 3 months [T1]) were asked to participate in a semi-structured interview, as well as patients who were invited but did not complete PROMs at both follow-up time points. Patients were asked for consent to the use of demographic and clinical data from the electronic medical records for the purpose of this evaluation study.

A convenience cohort of four healthcare professionals in various roles (nurse, resident internal medicine, fellow vascular medicine, and internist specialized in vascular medicine) who worked with PROM results at the outpatient clinic were interviewed about their experience with the use of PROMs. The same healthcare professionals were asked to complete the NoMAD questionnaire to assess the implementation process from their perspective.

### Data Collection

Semi-structured interviews were conducted by one researcher (CdJ) in the Dutch language. Questions were asked in a fixed order, according to an interview guide that was prepared for this evaluation study ( Table 2 ). Patients who had completed PROMs were interviewed on their experiences with the PROMs in practice, including their experiences with the completion of the questionnaires and their experiences during the outpatient clinic visit. Patients who had not completed PROMs after invitations at the two-time points were interviewed about their experiences around the PROMs and during their outpatient clinic visit too. There was no established relationship between the interviewer and the patients prior to the start of the interview. Demographic and clinical data were collected from the electronic medical records. Healthcare professionals were interviewed on their experiences with the use of PROMs in preparation for the patient appointment and during the appointment, and their perception of the value of the use of PROMs at the outpatient clinic. Field notes were made during all interviews.

**Table 2:** Guide for structured interviews with patients who completed PROMs, patients who did not complete PROMs, and professionals who worked with PROM results

Patients who completed PROMs	1. What is your experience with filling out the questionnaires? On a scale of 1 to 5 (1 negative, 5 positive)?
	2. How much time did you need to complete the questionnaires?
	3. The number of questions was a. too few, b. too many, c. just right
	4. The next three questions are about the questionnaires 1. Were any questions unclear? 2. Did you encounter questions that were not relevant to you? 3. Did you miss any questions?
	5. Did the care provider follow up on the responses you provided in the questionnaires? If so, how did you notice this? Do you feel that this contributed to your treatment? On a scale of 1 to 5 (1 none, 5 significantly)? If not, did you initiate a conversation about the questionnaires yourself?
	6. Did you experience that attention was paid to the symptoms and/or issues you wanted to discuss during the appointment? If not, did you initiate a conversation about the symptoms and/or issues you wanted to discuss during the appointment yourself?
	7. Did you feel prepared for the appointment after completing the questionnaires? On a scale of 1 to 5 (1 not at all, 5 very well prepared)?
	8. Do you have any suggestions for improvement?
Patients who did not complete PROMs	1. Did you start filling out the questionnaires, or were you unable to start the questionnaires?
	2. What caused you not to complete the questionnaires?
	3. What would have prompted you to fill out the questionnaires?
	4. Did you experience that attention was paid to the symptoms and/or issues you wanted to discuss during the appointment? If not, did you initiate a conversation about the symptoms and/or issues you wanted to discuss during the appointment yourself?
	5. Did you feel prepared for the appointment? On a scale of 1 to 5 (1 not at all, 5 very well prepared)?
Professionals	1. How many patients at your outpatient clinic have completed PROMs?
	2. What is your experience with the use of PROMs? On a scale of 1 to 5 (1 negative, 5 positive)?
	3. Did you use PROMs during preparation for the patient appointment and/or during the appointment? If so, how did you use the PROMs? Do you feel that this was of added value, and if so, in what way? Did you make a note in the medical records? What is your experience with the interpretation of PROM responses?
	4. What is your perception of the value of the use of PROMs at the outpatient clinic? And specifically, during the preparation for the patient appointment, and during the appointment? On a scale of 1 to 5 (1 none, 5 significant added value)?
	5. What could be improved about the PROMs, implementation, and/or use in practice?

Abbreviations: PROMs, patient-reported outcome measures.

In addition, to assess the implementation process from the perspective of involved healthcare professionals, the NoMAD questionnaire was used. This instrument was developed based on the normalization process theory (NPT) which explains the normalization of changes (a new intervention becoming part of normal practice) and was validated for the assessment of staff perceptions of implementation processes. 24 25 Four constructs proposed by the NPT are measured with the NoMAD instrument: coherence, cognitive participation, collective action, and reflexive monitoring. 25 26 In the current study, the Dutch translation of the NoMAD questionnaire was used. 27

### Data Analysis

Demographic variables of patients who were interviewed, completion rate, and quantitative data obtained with the interviews and NoMAD questionnaire were analyzed using descriptive statistics. The interviews were thematically analyzed. Themes were derived and identified from the data, and were described along with illustrative examples. All analyzes were performed using SPSS version 29.

## Results

### Completion Rate

From March to September 2023, 27 patients who had received follow-up for at least 3 months at the outpatient clinic (as identified per September 2023) received invitations to complete PROMs at the first (T0; 7 to 10 days after VTE diagnosis) and second-time point (T1; after 3 months). In response to the T0 invitation, PROMs were completed by 13/27 (48%) patients. At T1, 11 (41%) patients had completed the PROMs. PROMs were fully completed at both time points by five patients.

### Patients

Five consecutive patients who had completed PROMs at both time points were interviewed. Three patients who had not completed PROMs at any time point were interviewed as well. The eight interviewed patients (50% female) had a median age of 57 years. Five had been diagnosed with acute PE while three had experienced acute DVT (of the five patients who completed PROMs, four had experienced acute PE and one acute DVT; Table 3 ).

**Table 3:** Characteristics of the patients who completed PROMs and the patients who did not complete PROMs

Characteristics	Patients who completed PROMs ( *n* = 5)	Patients who did not complete PROMs ( *n* = 3)
Female ( *n* , %)	2 (40)	2 (67)
Age, in y (median, range)	54 (34–75)	59 (48–84)
Venous thromboembolic event ( *n* , %)		
–Acute pulmonary embolism	4 (80)	1 (33)
–Acute deep vein thrombosis	1 (20)	2 (67)

Abbreviations: PROMs, patient-reported outcome measures.

### Experiences of Patients with Completing PROMs

Patients who did complete PROMs at the T0 and T1 time points were asked about their experience with completion of the PROMs on a scale from 1 “negative” to 5 “positive”, and were neutral to positive (range: 3.0–4.5; two expressed 3.0 referring to neutral). A summary of the patients' experiences with the PROMs, illustrated with examples, is provided in Table 4 . Three of the five patients felt that all questions were clear, of whom one stated that the questions were “understandable for everyone”. However, one patient felt that questions were confusing and found it difficult to determine whether symptoms were due to the thrombosis or comorbidities. Four out of five patients expressed that the number of questions was too high; one patient stated not to remember the length of the questionnaires.

**Table 4:** Experiences with PROMs shared by patients who completed PROMs and professionals who worked with PROMs at the outpatient clinic

Theme	Shared experience
Patients' experiences	
Completion of the PROMs	–User-friendly –Filing out the questions via email works well –Questionnaires are very lengthy –A lot of similar or nearly identical questions, but phrased slightly differently –Neutral; no negative feeling, nor the feeling it helped me significantly
Relevance of the questions	–The questions also reveal things I would not have thought of –Some questions cause unease that I did not experience before, for instance, the question “were you afraid of being alone?” –Not all questions align with my perspective –There were questions that had nothing to do with the symptoms I experienced; I answered questions that had nothing to do with the veins/embolism –It felt like the questionnaires were designed for a senior individual, questions did not cover my daily activities
Purpose of the PROMs	–Makes you wonder why you are filling this out –In my view, these questions provide information, allowing the expert to learn more about the patient –It was confusing for me because I was in a whole process (due to other diseases), and it was not clear to me that the questionnaires were from the thrombosis outpatient clinic
Communication between patient and care provider, from the patient's perspective	
Follow up on responses to the questionnaires during the appointment	All five patients had experienced that the care provider did not follow up on the responses to the questionnaires during the appointment, but also none of them initiated a conversation about the questionnaires themselves; three patients did not feel the need to do so, and one patient did not know that the doctor was aware of the questionnaires.
Attention to symptoms and/or issues	–Pleasant experience, everything was discussed without me having to initiate anything –I was able to discuss everything –What was discussed during the appointment aligned well with what I wanted to know, I looked up things on the internet and could ask questions about that –Positive feeling with the doctor, who knew what was going on, it was a pleasant interaction –No, but I did not feel the need to start discussing other relevant matters, although there was room to do so
Preparation of the patient for the appointment	
Better prepared after completion of the PROMs	–I gained insights from some of the questions, there were questions where I thought “could that also be related,” or where I wanted to ask about during the appointment –Subconsciously, yes, something in the back of your mind
Not better prepared after completion of the PROMs	–I felt prepared regardless of the questionnaires –No, the questionnaires were more of an afterward realization, with no preparation or introduction for the conversation –No, but I did not feel the need to prepare for anything
Professionals' experiences	
Impression of the patient's well-being	–PROMs provide valuable insights, focusing on physical and social aspects, which help understand the patient's condition; I find the responses entrusted to be very useful –The PROMs are useful; you can read what is going on with the patient, the most helpful aspect is “questions for the doctor?” –During preparation for the consultation, I truly get an impression of how the patient is doing based on the PROMs
Direction of the conversation	–The PROMs provide insights that could guide the conversation –You can ask the patient to tell more about a specific topic
Value of use of PROMs	–PROMs allow to better help patients, to pay more attention to what is important to them –Based on the completed questionnaires, issues or complaints can be identified –PROMs help to not overlook something; something could become apparent when seeing the answers to the PROMs –You can measure the course over time in an objective manner; which is useful to evaluate if patients still have symptoms; this could be used to consider rehabilitation –You can come back to specific things from the questionnaire –The patient has thought about his/her health; this enhances efficiency –Health outcomes as experienced by the patient are neatly recorded –By asking more specific questions, I could save time –It would take a lot of time if every patient at the outpatient clinic would have filled out the PROMs
Application of the PROMs by professionals	
Preparation of the appointment	All four professionals used the PROMs during their preparation of the consultation. –Very useful –Looking at the results, at the colors, could help formulate questions –Interesting to look into the PROMs
During the appointment	Three professionals used the PROMs during the appointment. –During the appointment, the PROMs can be used as a tool to steer the conversation –Asking the patient, for example, to explore certain topics –Especially valuable at 3 months; then the PROMs provide insight into the course over time –Showing graphs (in the dashboard) to the patient, and using this as an entry point
Note in the medical records	–The responses to PROMs are implicitly part of my documentation –I made a summary and noted scores that I easily recognized –I documented the interpreted results, not the exact scores of the questionnaires –I interpreted the PROMs and made a note of that
Interpretation of the PROM responses	–Interpretation of the PROMs in the dashboard is good, the scores and colors are clear –Very straightforward –The overview works well, is functional, practical –The layout is a bit cluttered –The course over time is nicely visible –I opened the questionnaires to look into the questions in detail –It would be helpful to have (a range of) normal values displayed in addition to the colors –Sometimes unclear to what extent a patient is affected socially or physically

Abbreviations: PROMs, patient-reported outcome measures.

Note: Examples are based on responses from the patients and professionals.

### Preparation for the Outpatient Clinic Visit

Two patients indicated that completion of the PROMs added to the feeling of being prepared for the visit; one of them described that some questions made her think about her situation and what she wanted to ask about. Of the other patients, two did not feel prepared for the outpatient clinic visit despite completing the questionnaires, and one felt prepared regardless of the PROMs. On a scale from 1 “not at all prepared” to 5 “very well prepared”, two patients felt not prepared at all, one patient expressed a neutral stance, and one patient felt very well prepared (range: 1.0–5.0).

The three patients who did not complete PROMs reported feeling neutral to very well prepared for the outpatient clinic visit (range: 3.0–5.0).

### Experiences of Healthcare Professionals

The professionals reported that they had worked with PROM results in 2 to 15 patients who had completed the PROMs. On a scale from 1 “negative” to 5 “positive”, their experience with the use of PROMs was predominantly positive (range: 3.0–4.0; one expressed 3.0). They considerably valued the use of PROMs (range: 4.0–5.0; two expressed 5.0) and perceived additional value of PROMs both during the preparation for the patient appointment and during the appointment. The professionals' experiences with the PROMs are summarised in Table 4 .

### Communication Between Patient and Healthcare Professional

All five patients answered that the care provider did not follow up on all the responses to the questionnaires during the appointment, and one patient had been asked by the care provider if the PROMs had been received well. Despite this, four of the five patients felt that attention was paid to the symptoms and/or issues they wanted to discuss.

Two of the three patients who did not complete PROMs felt that attention was paid to the symptoms and/or issues they wanted to discuss during the appointment.

### Reasons to Not Complete PROMs

Of the patients who did not complete PROMs, one began filling out PROMs but paused during the questionnaires, and was unable to go back to continue with the remaining questions due to technical issues. For one patient, it was not clear how to answer the questionnaires. The third patient stated that she did not fill out PROMs because she did not feel the need to do so, as this was optional. Healthcare professionals noted that, in addition to patients who did not complete PROMs, some patients at the outpatient clinic had not received the invitations because they had not followed the complete care pathway, for instance when patients were referred from another hospital not directly after the VTE diagnosis.

### NoMAD Questionnaire

**Fig. 2:**
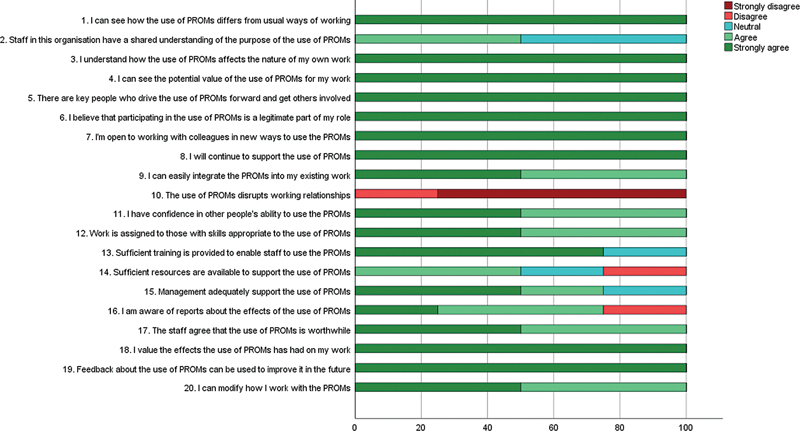
Frequency distribution of responses to the NoMAD questionnaire assessing the implementation process from the healthcare professional's perspective. The bars show the percentages of healthcare professionals reporting “strongly disagree”, “disagree”, “neutral”, “agree”, or “strongly agree” to each of the questions. Constructs: questions 1 to 4: coherence, questions 5 to 8: cognitive participation, questions 9 to 15: collective action, and questions 16 to 20: reflexive monitoring. NoMAD: normalization measure development, PROMs: patient-reported outcome measures.

Fig. 2 shows the responses to the NoMAD questionnaire, assessing the implementation process from the professionals' perspective. All four healthcare professionals strongly agreed with the potential value of the use of PROMs at the outpatient clinic and valued the effects that the use of PROMs had on their work. Also, they all stated to continue to support the use of PROMs, and all strongly believed that feedback about the use of PROMs can be used to improve its application in the future. They believed that key individuals play a crucial role in driving the use of PROMs and engaging others, and also considered participation in the use of PROMs as part of their own responsibilities (questions: 4–6, 8, and 18, 19).

Healthcare professionals expressed positive views regarding the integration of the PROMs into their work and felt that they could adapt their approach to using PROMs (questions: 9 and 20).

There was unanimous disagreement with the statement that the use of PROMs disrupts working relationships (question: 10).

Not all agreed that sufficient resources are available to support the use of PROMs and one of the healthcare professionals felt unaware of reports about the effects of the use of PROMs (questions: 14 and 16).

Furthermore, some healthcare professionals took a neutral stance on whether the training provided is sufficient to enable staff to use PROMs, whether management adequately supports the use of PROMs, and whether there is a shared understanding among staff regarding the purpose of PROMs (questions: 2, 13, and 15).

### Suggestions for Improvement

Patients and professionals were asked how the (use of) PROMs could be improved. Suggestions were shared to enhance the PROM completion rate and improve accessibility ( Table 5 ). Clarifying the purpose and relevance of PROMs in the e-mail with invitation link sent to patients, including the explanation that not all questions may be applicable to each individual, could enhance patient experiences and their willingness to complete the questionnaires. Also, the number of questions could be reduced. In addition, some patients indicated to have missed certain specific questions, for example, questions about the effectiveness of the medication and side effects, or about work, sports, and needs toward rehabilitation. Lastly, ensuring proper alignment of the outpatient appointments and measurement time points, and suggestions for improvement of technical aspects related to the digital PROMs application and to the dashboard facilitated by the digital tool were mentioned by professionals.

**Table 5:** Suggestions and considerations for improvement, shared by patients and healthcare professionals

Topic for improvement	Suggestions and considerations
Completion of the PROMs	–The option to fill out the questionnaires at the outpatient clinic would be convenient; for instance, completing PROMs on a tablet in the waiting room, potentially with the help of a volunteer (P, HCP) –Administering the questionnaires by phone (HCP)
Accessibility for patients	–Clear instructions on where the questionnaires can be filled out (P) –The possibility to resume filling out the questionnaires after pausing (P) –The PROMs are not accessible for non-Dutch speakers or individuals who cannot handle digital questionnaires (HCP)
Purpose of the PROMs and relevance to the patient	–Adding a sentence to the appointment letter, to announce that an invitation link will be sent (HCP) –In the instructions accompanying the invitation link, mention that the PROMs are sent from the thrombosis outpatient clinic (P) –In the instructions accompanying the invitation link, mention that the PROMs contain general inventory questions about health and functioning to get an impression of the patient's well-being as well as questions about symptoms or consequences related to the thrombotic event, which may not all be relevant or applicable to each individual (P) –Note that after filling out the questionnaires, the care provider has insight into the answers (P)
Content of the PROMs	
Shortening the questionnaires	–Reduce the question load (HCP) –Combining similar or slightly differently phrased questions (P; although complete validated questionnaires have been added to the PROMs set, considering scoring and interpretation of the responses)
Modifications to the PROMs	–Adding questions about the effectiveness of the medication and about side effects (P) –Adding questions about work and sports, what is needed in those areas, and about needs toward rehabilitation (P) –Adding free-text fields to the questionnaires, allowing for further elaboration (HCP) –Provide the option to fill in “not applicable” (P, HCP)
Accessibility for professionals	–All professionals who work with PROMs should have access to the dashboard (HCP)
Timing	–Timing of the PROMs measurements; attention to proper alignment between the invitation links and the appointments (HCP)
Digital PROMs application	
Technical aspects	–Manual activation/deactivation of PROMs invitations (HCP) –If possible, create a function that allows to transfer of an overview of PROM results directly into the medical records, to enhance visibility (HCP)
Interpretation of the PROM responses	–Cleaner layout (HCP) –Adding (ranges of) reference values in addition to the colors indicating normal/abnormal values (HCP)

Abbreviations: PROMs, patient-reported outcome measures.

Note: Suggested by: patient (P) and/or healthcare professional (HCP).

## Discussion

This first evaluation after the implementation of routine use of PROMs for VTE patients visiting our outpatient clinic revealed that both patients and healthcare professionals when asked about their experiences, felt neutral to positive about the use of PROMs. Notably, PROMs were completed by less than half the patients who received the invitation. Professionals perceived additional value of PROMs both during preparation for the patient appointment and during the appointment. Patients who completed the PROMs, however, indicated that their responses to the questionnaires were not always addressed during the appointment, but despite this, felt that the symptoms and/or issues they wanted to discuss had been paid attention, while patients who did not complete PROMs also felt that they had been given proper attention. For some patients, the PROMs enhanced the preparedness for the outpatient clinic visit, while others did not feel prepared for the visit despite completing the questionnaires or felt prepared regardless of the PROMs. The majority of the patients felt that the PROMs contained too many questions.

Implementation of PROMs into routine care comes with challenges. Web-based data entry may support PROMs completion and processing, by enabling to automatically incorporate the data into the electronic health records or other digital platforms that are designed to capture patient data. 28 Electronic data processing could also facilitate the interpretation of the PROM responses through analysis and (visual) presentation of the results, which could facilitate the use of PROM results by care providers in clinical decision-making. The completion of PROMs by patients requires (digital) literacy and skills. Also, not all questionnaires are available in multiple languages. Both the available resources and local context could affect the implementation success. 23 Moreover, the engagement of involved staff and dedicated personnel to coordinate the implementation process is essential for the integration of PROMs into routine care. 29 In our study, the involved healthcare professionals all felt committed to continue providing support to the use of PROMs.

The results of this early evaluation are encouraging, affirming the potential of routine use of PROMs for VTE patients, while key lessons can be learned that will benefit further implementation and application of PROMs in routine care. First, resources to increase and optimize the use of PROMs could be made available, including the potential to generate overviews of the distribution of PROM invitations, as well as the technical resources to support data processing and interpretation of the PROM responses. Second, patients should be better informed about the purpose and relevance of the questionnaires. Third, training and education on the application and interpretation of PROMs and their effects could improve healthcare professionals' ability to use the PROMs and enhance patients' experiences. One example would be to share the instruction to always discuss PROM results with the patient and follow up on responses to the questionnaires during the appointment. Lastly, reduction of the question load could improve the completion and use of PROMs.

The feasibility of implementation of other ICHOM standard sets has been demonstrated in several studies. 29 30 31 32 33 34 35 In a study evaluating the implementation of the ICHOM standard set for stroke, PROMs were considered relevant by patients, although they were found to have a limited understanding of the purpose of PROM assessment. 36 This is in line with our findings based on patients' experiences. Reported facilitators for successful implementation include the direct value of PROMs on individual patient care, professional education and feedback, and efforts to motivate patients to complete PROMs. 37 38 All professionals participating in the current study believed that feedback about the use of PROMs can indeed further improve its successful and meaningful application. Studies in the field of nephrology provided insights into the application of PROMs and guidance for optimal discussion of PROM results. 39 40 Both patients and healthcare professionals highlighted the importance of always discussing PROM results, with active participation of patients and a guiding role of professionals. Key enablers included a trustful relationship between the patient and care provider, a safe and private setting during a face-to-face consultation, an announcement of the discussion about PROM results during the appointment, and focusing on the most important topics during the consultation to deal with time constraints. These findings can be used for training of healthcare professionals.

The study has some limitations. First, the number of participants is small. As this was an evaluation study at a single academic hospital, performed a few months after implementation of the PROMs as part of routine care, we included as many patients who encountered the PROMs during follow-up at the outpatient clinic as were available. Consequently, our findings may not be generalizable to other hospitals or settings. We described the insights based on the first experiences of patients and healthcare professionals, but could not draw definitive conclusions due to the small sample size. Second, the patients who had completed PROMs at both follow-up time points could not accurately recall the time they spent completing the questionnaires. However, as patients indicated that the number of questions was too large, we still gained insight into their experience with the time burden associated with the completion of the PROMs.

Future studies are needed to assess how insights gained from the questionnaires are used in daily care, as well as to determine appropriate follow-up actions and evaluation in relation to specific PROM results, and their impact on outcomes such as quality of life.

## Conclusion

We gained insights based on the first experiences of patients and healthcare professionals with the use of PROMs in routine outpatient thrombosis care. PROMs were considered valuable by the healthcare professionals, and are believed to provide additional value during preparation for the visit to the outpatient clinic as well as during the visit. Patients, however, expressed that the PROMs contained too many questions and that their responses were not always addressed during the visit, but despite this, felt that they had been given proper attention. Some patients felt better prepared for the visit due to the completion of the PROMs, while others did not. The experiences and suggestions for improvement can be used to improve the application of PROMs in clinical practice and support further implementation of PROMs in daily thrombosis care.

## References

[1] American Heart Association Council on Peripheral Vascular Disease, Council on Clinical Cardiology, and Council on Cardiovascular and Stroke Nursing Kahn S R Comerota A J Cushman M The postthrombotic syndrome: evidence-based prevention, diagnosis, and treatment strategies: a scientific statement from the American Heart Association Circulation 2014 130 18 1636 1661 25246013 10.1161/CIR.0000000000000130

[2] Wolberg A S Rosendaal F R Weitz J I Venous thrombosis Nat Rev Dis Primers 2015 1 15006 27189130 10.1038/nrdp.2015.6

[3] Huisman M V Barco S Cannegieter S C Pulmonary embolism Nat Rev Dis Primers 2018 4 18028 29770793 10.1038/nrdp.2018.28

[4] Klok F A Mos I C Broek L Risk of arterial cardiovascular events in patients after pulmonary embolism Blood 2009 114 08 1484 1488 19549987 10.1182/blood-2009-05-220491

[5] Klok F A Zondag W van Kralingen K W Patient outcomes after acute pulmonary embolism. A pooled survival analysis of different adverse events Am J Respir Crit Care Med 2010 181 05 501 506 19965808 10.1164/rccm.200907-1141OC

[6] Klok F A van der Hulle T den Exter P L Lankeit M Huisman M V Konstantinides S The post-PE syndrome: a new concept for chronic complications of pulmonary embolism Blood Rev 2014 28 06 221 226 25168205 10.1016/j.blre.2014.07.003

[7] Barco S Mahmoudpour S H Valerio L Trends in mortality related to pulmonary embolism in the European Region, 2000-15: analysis of vital registration data from the WHO mortality database Lancet Respir Med 2020 8 03 277 287 31615719 10.1016/S2213-2600(19)30354-6

[8] FOCUS Investigators Valerio L Mavromanoli A C Barco S Chronic thromboembolic pulmonary hypertension and impairment after pulmonary embolism: the FOCUS study Eur Heart J 2022 43 36 3387 3398 35484821 10.1093/eurheartj/ehac206 PMC9492241

[9] Kahn S R Ducruet T Lamping D L Prospective evaluation of health-related quality of life in patients with deep venous thrombosis Arch Intern Med 2005 165 10 1173 1178 15911732 10.1001/archinte.165.10.1173

[10] Klok F A van Kralingen K W van Dijk A P Quality of life in long-term survivors of acute pulmonary embolism Chest 2010 138 06 1432 1440 20495104 10.1378/chest.09-2482

[11] Valerio L Barco S Jankowski M Quality of life 3 and 12 months following acute pulmonary embolism: analysis from a prospective multicenter cohort study Chest 2021 159 06 2428 2438 33548221 10.1016/j.chest.2021.01.071

[12] Braekkan S K Grosse S D Okoroh E M Venous thromboembolism and subsequent permanent work-related disability J Thromb Haemost 2016 14 10 1978 1987 27411161 10.1111/jth.13411 PMC5083219

[13] Jørgensen H Horváth-Puhó E Laugesen K Brækkan S Hansen J B Sørensen H T Risk of a permanent work-related disability pension after incident venous thromboembolism in Denmark: a population-based cohort study PLoS Med 2021 18 08 e1003770 34464405 10.1371/journal.pmed.1003770 PMC8443033

[14] Hunter R Noble S Lewis S Bennett P Long-term psychosocial impact of venous thromboembolism: a qualitative study in the community BMJ Open 2019 9 02 e024805 10.1136/bmjopen-2018-024805 PMC6377529 30782919

[15] Kirchberger I Ruile S Linseisen J Haberl S Meisinger C Berghaus T M The lived experience with pulmonary embolism: a qualitative study using focus groups Respir Med 2020 167 105978 32421544 10.1016/j.rmed.2020.105978

[16] Tran A Redley M de Wit K The psychological impact of pulmonary embolism: a mixed-methods study Res Pract Thromb Haemost 2021 5 02 301 307 33733029 10.1002/rth2.12484 PMC7938621

[17] Jørgensen H Horváth-Puhó E Laugesen K Brækkan S K Hansen J B Sørensen H T Venous thromboembolism and risk of depression: a population-based cohort study J Thromb Haemost 2023 21 04 953 962 36696217 10.1016/j.jtha.2022.12.006

[18] Dawson J Doll H Fitzpatrick R Jenkinson C Carr A J The routine use of patient reported outcome measures in healthcare settings BMJ 2010 340 c186 20083546 10.1136/bmj.c186

[19] Black N Patient reported outcome measures could help transform healthcare BMJ 2013 346 f167 23358487 10.1136/bmj.f167

[20] Churruca K Pomare C Ellis L A Patient-reported outcome measures (PROMs): A review of generic and condition-specific measures and a discussion of trends and issues Health Expect 2021 24 04 1015 1024 33949755 10.1111/hex.13254 PMC8369118

[21] Rotenstein L S Huckman R S Wagle N W Making patients and doctors happier - the potential of patient-reported outcomes N Engl J Med 2017 377 14 1309 1312 28976860 10.1056/NEJMp1707537

[22] de Jong C MM Rosovsky R P Klok F A Outcomes of venous thromboembolism care: future directions J Thromb Haemost 2023 21 05 1082 1089 36863565 10.1016/j.jtha.2023.02.015

[23] Gwozdz A M de Jong C MM Fialho L S Development of an international standard set of outcome measures for patients with venous thromboembolism: an International Consortium for Health Outcomes Measurement consensus recommendation Lancet Haematol 2022 9 09 e698 e706 36055334 10.1016/S2352-3026(22)00215-0

[24] Rapley T Girling M Mair F S Improving the normalization of complex interventions: part 1 - development of the NoMAD instrument for assessing implementation work based on normalization process theory (NPT) BMC Med Res Methodol 2018 18 01 133 30442093 10.1186/s12874-018-0590-y PMC6238361

[25] Finch T L Girling M May C R Improving the normalization of complex interventions: part 2 - validation of the NoMAD instrument for assessing implementation work based on normalization process theory (NPT) BMC Med Res Methodol 2018 18 01 135 30442094 10.1186/s12874-018-0591-x PMC6238372

[26] May C R Mair F Finch T Development of a theory of implementation and integration: normalization process theory Implement Sci 2009 4 29 19460163 10.1186/1748-5908-4-29 PMC2693517

[27] Vis C Ruwaard J Finch T Toward an objective assessment of implementation processes for innovations in health care: psychometric evaluation of the normalization measure development (NoMAD) questionnaire among mental health care professionals J Med Internet Res 2019 21 02 e12376 30785402 10.2196/12376 PMC6401675

[28] de Jong C MM de Wit K Black S A Use of patient-reported outcome measures in patients with venous thromboembolism: communication from the ISTH SSC subcommittee on predictive and diagnostic variables in thrombotic disease J Thromb Haemost 2023 21 10 2953 2962 37394119 10.1016/j.jtha.2023.06.023

[29] Ackerman I N Cavka B Lippa J Bucknill A The feasibility of implementing the ICHOM Standard set for hip and knee osteoarthritis: a mixed-methods evaluation in public and private hospital settings J Patient Rep Outcomes 2018 2 32 30148249 10.1186/s41687-018-0062-5 PMC6091617

[30] Depla A L Ernst-Smelt H E Poels M Crombag N M Franx A Bekker M N A feasibility study of implementing a patient-centered outcome set for pregnancy and childbirth Health Sci Rep 2020 3 03 e168 32607452 10.1002/hsr2.168 PMC7317300

[31] Niazi S K Spaulding A Vargas E Feasibility study of three-phase implementation of international consortium for health outcomes measurement depression and anxiety standard set in an outpatient consultation-liaison psychiatry practice Psychosomatics 2020 61 01 8 18 31648776 10.1016/j.psym.2019.08.006

[32] Queirós L Redondo P França M Implementing ICHOM standard set for cataract surgery at IPO-Porto (Portugal): clinical outcomes, quality of life and costs BMC Ophthalmol 2021 21 01 119 33673817 10.1186/s12886-021-01887-6 PMC7936410

[33] Al-Shammari I Roa L Yorlets R R Implementation of an international standardized set of outcome indicators in pregnancy and childbirth in Kenya: utilizing mobile technology to collect patient-reported outcomes PLoS One 2019 14 10 e0222978 31618249 10.1371/journal.pone.0222978 PMC6795527

[34] Bittar P G Carlson A R Mabie-DeRuyter A Marcus J R Allori A C Implementation of a standardized data-collection system for comprehensive appraisal of cleft care Cleft Palate Craniofac J 2018 55 10 1382 1390 29561717 10.1177/1055665618764952

[35] Depla A L Pluut B Lamain-de Ruiter M PROMs and PREMs in routine perinatal care: mixed methods evaluation of their implementation into integrated obstetric care networks J Patient Rep Outcomes 2023 7 01 26 36894797 10.1186/s41687-023-00568-w PMC9998006

[36] Lebherz L Fraune E Thomalla G Implementability of collecting patient-reported outcome data in stroke unit care - a qualitative study BMC Health Serv Res 2022 22 01 346 35292028 10.1186/s12913-022-07722-y PMC8925160

[37] Depla A L Crombag N M Franx A Bekker M N Implementation of a standard outcome set in perinatal care: a qualitative analysis of barriers and facilitators from all stakeholder perspectives BMC Health Serv Res 2021 21 01 113 33530989 10.1186/s12913-021-06121-z PMC7852077

[38] Chen A Väyrynen K Leskelä R L A qualitative study on professionals' attitudes and views towards the introduction of patient reported measures into public maternity care pathway BMC Health Serv Res 2021 21 01 645 34217284 10.1186/s12913-021-06658-z PMC8254939

[39] van der Willik E M Hemmelder M H Bart H AJ Routinely measuring symptom burden and health-related quality of life in dialysis patients: first results from the Dutch registry of patient-reported outcome measures Clin Kidney J 2020 14 06 1535 1544 34285801 10.1093/ckj/sfz192 PMC8286800

[40] van der Willik E M Milders J Bart J AJ Discussing results of patient-reported outcome measures (PROMs) between patients and healthcare professionals in routine dialysis care: a qualitative study BMJ Open 2022 12 11 e067044 10.1136/bmjopen-2022-067044 PMC9677037 36396312

